# Synthesis of Multifunctional Mn_3_O_4_-Ag_2_S Janus Nanoparticles for Enhanced T_1_-Magnetic Resonance Imaging and Photo-Induced Tumor Therapy

**DOI:** 10.3390/s23218930

**Published:** 2023-11-02

**Authors:** Yuguang Lu, Yuling Wu, Zhe Tang, Yike Hou, Mingyue Cui, Shuqi Huang, Binghua Long, Zhangsen Yu, Muhammad Zubair Iqbal, Xiangdong Kong

**Affiliations:** 1 Institute of Smart Biomedical Materials, School of Materials Science and Engineering, Zhejiang Sci-Tech University, Hangzhou 310018, China; 2 Laboratory of Nanomedicine, Medical Science Research Center, School of Medicine, Shaoxing University, Shaoxing 312000, China; yzs@usx.edu.cn

**Keywords:** ultra-small Janus nanoparticles, cancer theranostics, T_1_ contrast agents, magnetic resonance imaging, photo-responsive materials

## Abstract

The global burden of cancer is increasing rapidly, and nanomedicine offers promising prospects for enhancing the life expectancy of cancer patients. Janus nanoparticles (JNPs) have garnered considerable attention due to their asymmetric geometry, enabling multifunctionality in drug delivery and theranostics. However, achieving precise control over the self-assembly of JNPs in solution at the nanoscale level poses significant challenges. Herein, a low-temperature reversed-phase microemulsion system was used to obtain homogenous Mn_3_O_4_-Ag_2_S JNPs, which showed significant potential in cancer theranostics. Structural characterization revealed that the Ag_2_S (5–10 nm) part was uniformly deposited on a specific surface of Mn_3_O_4_ to form a Mn_3_O_4_-Ag_2_S Janus morphology. Compared to the single-component Mn_3_O_4_ and Ag_2_S particles, the fabricated Mn_3_O_4_-Ag_2_S JNPs exhibited satisfactory biocompatibility and therapeutic performance. Novel diagnostic and therapeutic nanoplatforms can be guided using the magnetic component in JNPs, which is revealed as an excellent T_1_ contrast enhancement agent in magnetic resonance imaging (MRI) with multiple functions, such as photo-induced regulation of the tumor microenvironment via producing reactive oxygen species and second near-infrared region (NIR-II) photothermal excitation for in vitro tumor-killing effects. The prime antibacterial and promising theranostics results demonstrate the extensive potential of the designed photo-responsive Mn_3_O_4_-Ag_2_S JNPs for biomedical applications.

## 1. Introduction

Cancer is universally recognized as the prime cause of morbidity and mortality globally [1]. An accurate cancer diagnosis is crucial for selecting appropriate and effective treatment options, such as surgery [2], radiotherapy [3,4,5], and chemotherapy [6,7,8], as each type of cancer requires a specific treatment. However, traditional cancer therapies may lead to serious side effects including cancer recurrence. Particularly, irreversible damage to healthy cells and emotional distress resulting from conventional non-specific treatments limits the survival rate [9]. Early precise diagnosis, improvement of drug delivery efficiency, and reduction of side effects of treatment have become the challenges of cancer diagnosis and treatment. Theranostics combines diagnosis and drug treatment, simultaneously providing both diagnosis and treatment to patients, which is a trend for future personalized medical development [10,11,12]. The past few decades have witnessed the rise of nanotechnology, providing new opportunities to tackle the challenges of clinical cancer diagnosis and treatment [13,14,15,16].

In the field of nanomaterials, Janus nanoparticles (JNPs) have garnered attention due to the intricate process of controlling their synthesis in solution [17,18]. The amalgamation of two distinct chemical natures poses a challenge as it may alter the original properties of nanoparticles, which are highly sought after for various applications in materials science, particularly in the biomedical field. Due to their anisotropic nature and differing physicochemical properties, multiple functionalities and ligands can be attached to the surface of JNPs to treat diseases [19,20,21,22,23]. Despite significant efforts by researchers to facilitate the self-assembly of Janus at the nanoscale level, it remains a challenging task, as evidenced by various reports [24,25,26,27]. Notably, the Janus structure enables the theranostic function, which combines diagnostic and therapeutic modalities [28,29,30,31]. Molecular imaging has contributed significantly to early disease detection and real-time monitoring of therapeutic outcomes in diagnosis. Among the various imaging techniques, magnetic resonance imaging (MRI) stands out for its high sensitivity, selectivity, non-interference, multi-plane scans, and localization ability without the risk of harmful ionizing radiation [32,33,34].

In recent years, manganese-based nanoparticles have become important T_1_-weighted contrast agents in MRI due to their high lateral relationship time and biocompatibility compared to clinical Gd-based chelates, which have serious side effects, such as dizziness, nausea, irreversible chronic nephropathy, and nephrogenic systemic fibrosis [35]. Moreover, various biocompatible polymers have been used to modify the surface of Mn-based NPs to enhance their biocompatibility. Based on its sensitivity to the acidic tumor microenvironment, Mn from Mn_3_O_4_ exhibits a mixture of +2 and +3 valence states, and the high-valent Mn ions can be reduced by glutathione (GSH) in the tumor microenvironment to enhance theranostic ability. Therefore, Mn-based nanoparticles have become the focus of current research, mainly as low-toxicity T_1_-weighted MRI contrast agents and antitumor agents [36,37].

Photothermal therapy (PTT), a noninvasive approach for eliminating solid tumors, has attained clinical attention in phototherapy applications. It has unique merits, including high spatiotemporal controllability [38], reduced side effects [39], and minimal-level invasiveness [40]. In addition, phototherapeutic agents have been shown to have novel properties for overcoming the deficiencies and limitations of traditional phototherapies, such as low efficiency and shallow light penetration into biological tissues [41,42]. The regulable size and morphology of nanomaterials result in high penetration and long retention effects in solid tumors, quickly and conveniently crossing the vascular wall to enrich tumor tissues and passively targeting the tumor area to achieve efficient diagnostic and therapeutic efficacy [43,44]. Over the past decades, various nanomaterials, such as carbon nanotubes and gold-based, silver-based, and copper-based nanomaterials, have been developed for PTT. Among them, Ag_2_S nanoparticles possessed promising responses in the second near-infrared region (NIR-II) window for deep tumor PTT [45,46,47].

**Scheme 1:**
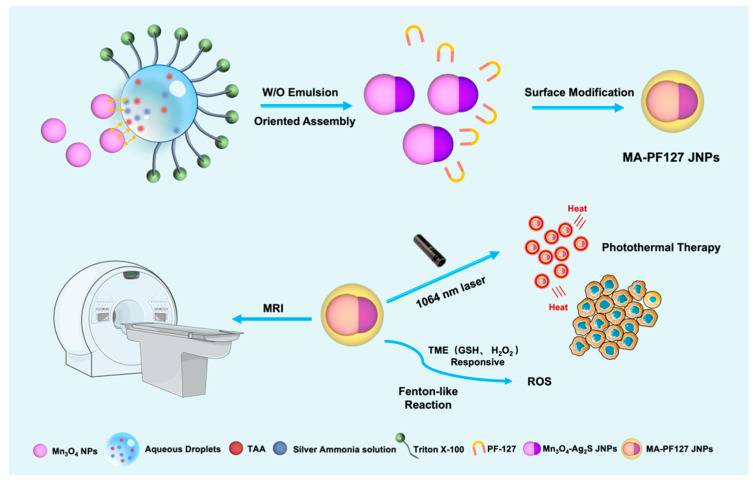
Synthetic route of the Mn_3_O_4_-Ag_2_S-PF127 JNPs and the potential capacity for MRI-guided photo-responsive cancer theranostics.

Through acknowledging the significance of theranostics, a novel Janus nanostructure comprising Mn_3_O_4_-Ag_2_S was designed and synthesized using the reverse microemulsion method for magnetic resonance imaging, photothermal therapy, and antibacterial applications (Scheme 1). Mn^2+^ ions can react with H_2_O_2_ in a microtumor environment with GSH as the trigger to generate reactive oxygen species (ROS) via a Fenton-like reaction and are also confirmed to be strong T_1_-MRI agents for cancer imaging. Additionally, the Ag_2_S component of the Janus structure plays a crucial role in high-efficiency NIR-II deep photothermal therapy and as antimicrobial agents. The synthesis approach presented in this study could offer a novel template to fabricate other Janus-type metal-based nanoparticles, possibly constructing a theranostic platform for multiple biomedical applications.

## 2. Materials and Methods

### 2.1. Materials

Manganese (II) Acetate Tetrahydrate (C_4_H_14_MnO_8_, Adamas, Shanghai, China), xylene (>99%, Greagent, Shanghai, China), oleylamine (>90%, Adamas, Shanghai, China), oleic acid (95%, Adamas, Shanghai, China), Triton X-100 (AR, Rhawn, Shanghai, China), Pluronic^®^ F-127 [poly (ethylene oxide)-poly(propylene oxide)-poly(ethylene oxide), PEO-PPO-PEO](PF127, General-reagent, Shanghai, China), Dulbecco’s modified Eagle medium (DMEM, high glucose, Gibco, C11995, Hangzhou, China), fetal bovine serum (FBS, Hyclone, Logan, UT, USA, SH30070.03), 3-(4,5-dimethylthiazol-2-yl)-2,5-diphenyl tetrazolium bromide (MTT, Beyotime, Shanghai, China), and phosphate buffer saline (PBS, Hyclone, Logan, UT, USA, SH30256.FS). Ethanol and cyclohexane (Shuanglin Chemicals, Hangzhou, China).

### 2.2. Synthesis of Mn_3_O_4_ Nanoparticles

Briefly, 0.17 g of manganese(II) acetate powder, 1.6 mL of oleic acid, and 2.775 mL of oleylamine were dissolved in 15 mL of xylene in an air atmosphere. The mixture was stirred for 10 min before heating to 90 °C, 1 mL of DI water was injected into the solution under vigorous stirring, and the resulting solution was maintained at 90 °C for 3 h. Ethanol was added to obtain a precipitate, and the final powdery form was collected via centrifugation. The synthesized nanocrystals were well-dispersed in cyclohexane.

### 2.3. Fabrication of Mn_3_O_4_-Ag_2_S JNPs

Triton X-100 (4 mL) and oleylamine (10 mL) were mixed via magnetic stirring in a beaker for 20 min. A total of 500 μL Mn_3_O_4_ (20 mg mL^−1^) nanoparticles in cyclohexane was added to the above mixture. Afterward, 500 μL of silver ammonia solution (32 mg mL^−1^) was added under vigorous stirring. After 3 h, 500 μL of a saturated thioacetamide solution was added for 24 h at room temperature. The resulting compound was washed thrice with distilled ethanol and purified via centrifugation at 7000× *g* rpm.

### 2.4. Preparation of Mn_3_O_4_-Ag_2_S-PF127 JNPs

Primarily, 800 mg of PF127 powder was dissolved in 25 mL of deionized water to form a transparent solution. The above solution was heated to 80 °C to evaporate the organic part. An aqueous solution of PF127-coated Mn_3_O_4_-Ag_2_S Janus nanoparticles (MA-PF127 JNPs) was obtained via washing with centrifuges and redispersing in deionized water.

### 2.5. Characterizations

Transmission electron microscopy (TEM) images and energy dispersive spectroscopy (EDS) data were acquired using a JEM-1400Flash instrument operated at 80 kV (Tokyo, Japan). Scanning transmission electron microscopy (STEM), EDS elemental mapping, and EELS curve analyses were conducted using Talos F200x (Thermo Fisher Scientific, Waltham, MA, USA). Zeta potential determinations and hydrodynamic diameters were investigated using a Malvern Zeta sizer Nano ZS instrument (Malvern, UK). The UV–vis absorption spectra were measured using a P7 double-beam UV–visible spectrophotometer (Shanghai, China). X-ray diffraction (XRD) patterns were obtained using a Bruker D8 X-ray diffractometer (Karlsruhe, Germany). Laser irradiation was performed using a diode infrared laser module (1064 nm, Changchun Laser Technology, China). Inductively coupled plasma mass spectrometry (ICP-MS) was conducted using an Optima 2100 instrument from PerkinElmer (Waltham, MA, USA).

### 2.6. Measurement of Dissolved Oxygen

A certain amount of the MA-PF127 JNPs was incubated with 10 mL of deionized water containing H_2_O_2_ (100 mM) at room temperature. The concentration of dissolved oxygen (O_2_) was continuously monitored using a dissolved oxygen meter.

### 2.7. Chemodynamic Activity of Mn_3_O_4_-Ag_2_S-PF127 JNPs

ROS production was quantitatively analyzed based on the catalytic properties of the MA-PF127 NPs through methylene blue (MB) degradation. Specifically, the absorbance at a wavelength of 644 nm from the MB solution (25 mg L^−1^) was measured at different pH (7.4 or 6.5), and concentrations of GSH (0, 1, 2.5, 5, and 10 mM). Measurements were taken before and after adding a specified amount of the MA-PF127 NPs, and the experiment duration was 3 h.

### 2.8. MRI and Relaxation Properties

The MRI and relaxation times (longitudinal (T_1_) and transverse (T_2_) relaxation times) of the MA-PF127 JNPs dispersed in deionized water with different concentrations based on Mn equivalent (0.14, 0.28, 0.57, 1.09, and 2.18 mM) were analyzed using a 0.55 T MRI scanner (Shanghai, Niumag Corporation) at 37 °C. The relaxivity values (r_1_ and r_2_) were calculated via performing linear fitting of the slopes of 1/T_1_ (s^−1^) and 1/T_2_ (s^−1^) versus the Mn concentration (mM). The measurement conditions were as follows: T_1_-weighted sequence, multi-slice spin echo (MSE): a repetition time (TR) of 300 ms, echo time (TE) of 18 ms, FOV = 100 mm × 100 mm, thickness = 3.0 mm, 0.55 T, 37.0 °C. The parameters adopted were as follows: a spin-echo sequence: a repetition time (TR) of 2000 ms, echo time (TE) of 50 ms, FOV = 100 mm × 100 mm, thickness = 3.0 mm, 0.55 T, 37.0 °C.

### 2.9. Photothermal Effect of Mn_3_O_4_-Ag_2_S-PF127 JNPs

Photothermal conversion efficiency was evaluated through irradiating 200 mL aqueous dispersions of the MA-PF127 NPs at distinct concentrations (0, 25, 50, and 75 μg mL^−1^ based on Ag equivalent) under a 1064 nm laser with a power density of 1.0 W cm^−2^ for 10 min. After that, 200 mL of the MA-PF127 NPs (75 μg mL^−1^ based on Ag) was irradiated under a 1064 nm laser with varying power densities (1.0, 1.2, and 1.5 W cm^−2^) for 10 min. Temperature changes were recorded in real-time using an infrared thermal imager (FLIR, SC300, Arlington). The photothermal stability of the MA-PF127 JNPs was determined via subjecting the sample to laser irradiation followed by a cooling period of 10 min. The above process was repeated three times.

### 2.10. Cell Culture

The biocompatibility of MA-PF127 was validated through selecting Human Umbilical Vein Endothelial Cells (HUVEC) and a mouse breast cancer cell line (4T1) for cellular experiments. HUVEC and 4T1 cells were cultured in DMEM medium supplemented with 10% Fetal Bovine Serum (FBS) and 1% penicillin/streptomycin (PS) at 37 °C under a 5% carbon dioxide atmosphere. The cells were seeded in a 96-well plate at a density of 1 × 10^4^ cells per well and incubated with varying concentrations (0, 1, 2.5, 5, 10, 20, 30, 40, and 50 μg mL^−1^ of Mn equivalent) of the MA-PF127 NPs dispersed in DMEM for 24 h. The viability of the cells in each well was assessed using the standard MTT assay.

### 2.11. In Vitro Photothermal Therapy

The in vitro photothermal properties of the MA-PF127 JNPs were demonstrated through the co-incubation of the material with the murine breast carcinoma (4T1) cells and cell survival after laser irradiation. The cells were seeded in a 96-well plate, cultivated for 24 h, and then cultivated with DMEM-diluted MA-PF127 JNPs at different concentrations (0, 1, 2.5, 5, 10, 20, 30, 40, and 50 μg mL^−1^ of Mn) for 12 h. The cells were washed with PBS three times and seeded with a fresh culture medium, whereafter it was exposed to 1064 nm laser irradiation for 10 min at an intensity of 1.2 W cm^−2^. After incubation for another 12 h, cell viability was evaluated using the MTT assay.

### 2.12. Bacterial Culture and Antibacterial Assay

The liquid medium used for bacterial culture and dilution in this study was Luria–Bertani (LB) broth. LB broth was prepared via dissolving trypsin (1 g), NaCl (1 g), and yeast extract (0.5 g) in 100 mL of deionized water. To obtain the LB agar medium, an additional 2 g of agar powder was added to the LB liquid medium. Both the LB broth and LB agar medium were sterilized at 120 °C for 30 min.

Monocolonies of *S. aureus* and *E. coli* cultured on LB agar plates were transferred to centrifuge tubes containing 10 mL of the LB medium, and the centrifuge tubes were placed on a shaker at constant temperature and shaken at 37 °C for overnight incubation.. The obtained bacterial broth was diluted in the LB medium, and the broth with an absorbance of 0.1 AU at 600 nm was measured using a UV spectrophotometer and diluted tenfold in the LB medium. Different concentrations of material dispersed in the LB medium containing 50 μL of bacteria (0, 1, 2.5, 5, 10, 20, 30, 40, and 50 μg mL^−1^ of Mn equivalent) were added to 96-well plates and incubated in a thermostatically shaken incubator (37 °C, 160 rpm) for 24 h, and enzyme markers were utilized to measure the absorbance at 600 nm. For the bacterial spread plate method, the bacteria incubated with the material for 24 h were diluted to 1 × 10^5^ times using the LB medium. The bacterial solution (0.1 mL) was added to the center of the LB solid medium, evenly spread on its surface using a spreading rod sterilized in alcohol, and incubated overnight in a constant-temperature shaking incubator (37 °C, 160 rpm).

## 3. Results and Discussions

### 3.1. Synthesis and Characterizations of Mn_3_O_4_-Ag_2_S JNPs

**Figure 1:**
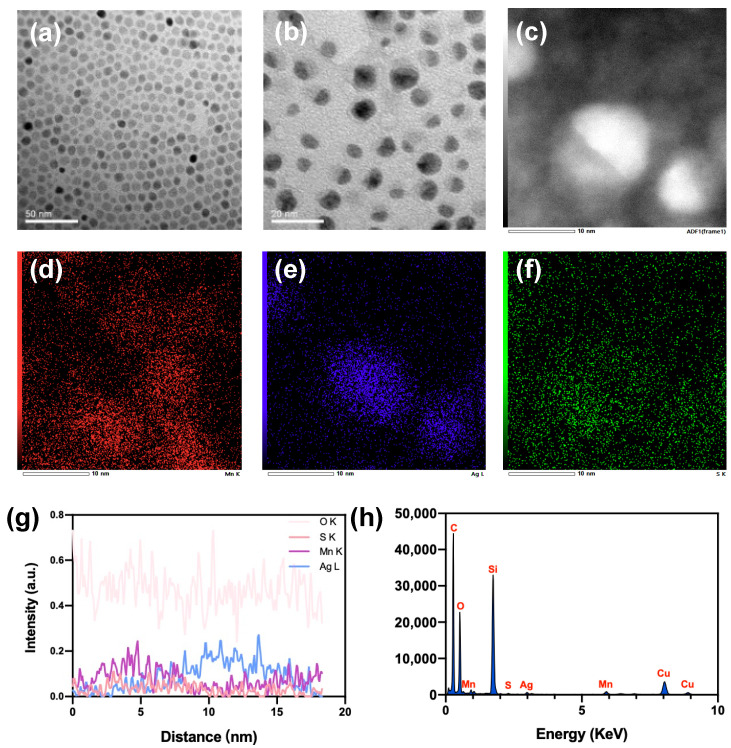
The structural characterization of Mn_3_O_4_ and the Mn_3_O_4_-Ag_2_S JNPs. (**a**,**b**) Magnified TEM images of Mn_3_O_4_ and the Mn_3_O_4_-Ag_2_S NPs. (**c**) HAADF-STEM. (**d**–**f**) Elemental mapping. (**g**) EELS line scan. (**h**) EDS spectra of the as-synthesized Mn_3_O_4_-Ag_2_S JNPs.

Initially, homogenous Mn_3_O_4_ nanocrystals were prepared using a simple solvothermal method. Next, the reverse microemulsion method was applied to fabricate the Mn_3_O_4_-Ag_2_S JNPs. In the first step, oleylamine and Triton X-100 were first mixed, and then Mn_3_O_4_ nanoparticles dispersed in cyclohexane were added under continuous stirring. Secondly, a reversed-phase microemulsion system was formed through precisely injecting aqueous droplets containing silver ammonia solution into the reaction system. A transparent yellow reverse microemulsion was formed via mixing both solutions for 3 h. Finally, the saturated TAA solution was added for 24 h to produce Mn_3_O_4_-Ag_2_S JNPs. The externally applied stirring force facilitated the orientation and assembly of tiny droplets containing the Ag_2_S precursor component on the monoside of the Mn_3_O_4_ nanoparticles in a reversed microemulsion solution for sufficient time, eventually leading to the formation of the Mn_3_O_4_-Ag_2_S JNPs (Scheme 1). A transmission electron microscope (TEM) was employed to investigate the structural features of the prepared materials. Figure 1a shows the excellent dispersion of as-synthesized Mn_3_O_4_ nanocrystals with an average diameter of 9 nm. The TEM images of the Mn_3_O_4_-Ag_2_S JNPs (Figure 1b) indicate that the Ag_2_S (5–10 nm) part was uniformly deposited on a specific surface of Mn_3_O_4_ to form the Janus morphology. The structure of the Mn_3_O_4_-Ag_2_S JNPs was further characterized via high-angle annular dark-field scanning transmission electron microscopy (HAADF-SETM) and related element mapping (Figure 1d–f). The formation of JNPs validated from Mn, Ag, and S elements through the distinct luminance contrast of Mn (red), Ag (blue), and S (green) represents the specific atomic density of Mn_3_O_4_ and Ag_2_S as JNPs. To confirm the synthesized Mn_3_O_4_-Ag_2_S JNPs, electron energy loss spectroscopy (EELS) line-scanning (Figure 1g) was conducted to analyze the distribution of the elemental signals along the axis of the JNPs. The results revealed that the Mn element is located at a distance of 0–10 nm from the Ag and S elements, which are positioned within the 8–15 nm range. The overlapping regions at 5 nm and 8 nm signify the intimate combination of Mn_3_O_4_ nanocrystals with the Ag_2_S component, facilitating mutual ion diffusion through the interface of these two compositions. Energy-dispersive X-ray spectroscopy (EDS) was utilized to qualitatively validate the elemental composition of the synthesized Janus-type nanomaterials, as shown in Figure 1h. The successful fabrication of the Mn_3_O_4_-Ag_2_S JNPs was confirmed via the detection of Mn, Ag, and S.

**Figure 2:**
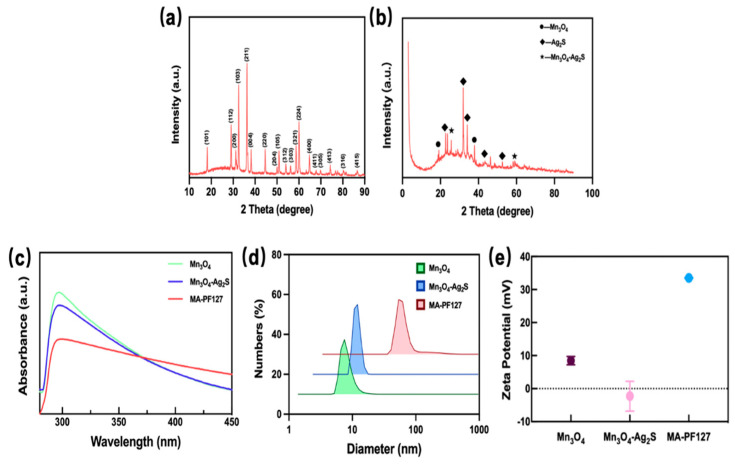
Material characterization. The powder XRD pattern of the prepared Mn_3_O_4_ (**a**) and Mn_3_O_4_-Ag_2_S nanoparticles (**b**). (**c**) UV-vis absorption spectra. (**d**) Size distribution histogram. (**e**) Zeta potential of the fabricated Mn_3_O_4_, Mn_3_O_4_-Ag_2_S, and MA-PF127 nanoparticles.

To observe the composite nature at the crystal level, X-ray diffraction (XRD) patterns were examined for the Mn_3_O_4_ NPs (Figure 2a) and Mn_3_O_4_-Ag_2_S NPs (Figure 2b). The XRD diffraction peaks corresponding to Mn_3_O_4_ (JCPDS#77-0435) and Ag_2_S (JCPDS#14-0072) verify the formation and high crystallinity of MAJNPs. Pluronic^®^ F-127 (PF127), a triblock copolymer approved by the U.S. Food and Drug Administration (FDA), was utilized for surface modification of the prepared JNPs to enhance biocompatibility.

Moreover, the absorption of the prepared samples was measured using ultraviolet-visible (UV–vis) spectroscopy. Figure 2c clearly demonstrates a blue shift in the maximum absorption wavelengths of MA and MA-PF127 compared to that of Mn_3_O_4_ dispersed in cyclohexane due to the effect of the absorption in the LSPR band and lower bands of silver sulfide (Ag_2_S). Simultaneously, the half-peak width becomes larger, and the absorption intensity decreases, proving the introduction of silver sulfide (Ag_2_S) and the coating of polymer PF127. Moreover, the significant changes in the sizes and zeta potentials of the synthesized Mn_3_O_4_, MA, and MA-PF127 NPs indicated the successful introduction of silver sulfide (Ag_2_S) and the surface modification of PF127 (Figure 2d,e). The hydrodynamic sizes of the synthesized materials are 7.55 nm, 12.18 nm, and 58.8 nm, respectively, further justifying the homogeneity of the nanoparticles. The zeta potentials of the Mn_3_O_4_, MA, and MA-PF127 NPs are 8.47, -2.28, and 33.5, which also proves the successful modification and excellent aqueous dispersion of the materials. In addition, the results from inductively coupled plasma mass spectrometry (ICP-MS) shows that the concentration of Mn in the MA-PF127 JNPs solution was 100 μg mL^−1^ and the concentration of Ag was 75 μg mL^−1^.

### 3.2. Fenton-like Properties of Mn_3_O_4_-Ag_2_S JNPs

The characteristics of the tumor microenvironment, such as hypoxia, acidity, and high glutathione (GSH)/H_2_O_2_ expression, limit photodynamic therapy. However, the Fenton-like activity of the manganese-based nanomaterials, in addition to being used for diagnostic applications, can improve the tumor’s lack of oxygen environment via catalyzing the generation of oxygen from overexpressed hydrogen peroxide in acidic environments as well as via oxidizing GSH to GSSH, promoting the generation of reactive oxygen species and improving the efficiency of related therapeutic tools. To explore the chemokinetic activity of the synthesized MA-PF127 nanomaterials, we performed oxygen and reactive oxygen generation assays under tumor microenvironment simulation conditions to verify the parallel effect of the manganese-based fraction of the material.

**Figure 3:**
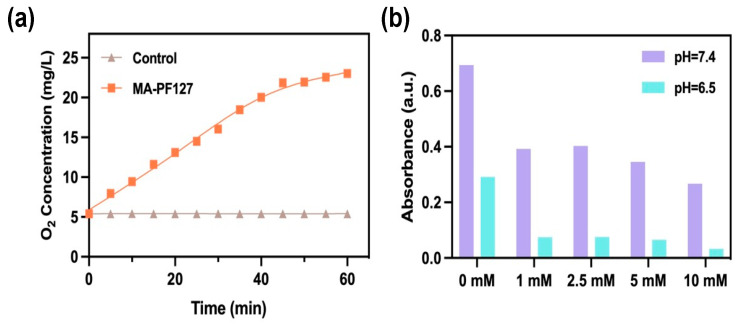
(**a**) Time-dependent evolution of dissolved oxygen concentration measured in solutions of H_2_O_2_ (100 mM) and the MA-PF127 NPs + H_2_O_2_ (100 mM). (**b**) UV-vis absorption of MB after degradation using the MA-PF127 NPs treated with various amounts of GSH at pH 7.4 and pH 6.5.

As shown in Figure 3a, through testing the values of dissolved oxygen in water containing a certain concentration of hydrogen peroxide under oil-sealed conditions, it can be seen that the dissolved oxygen content in the set control group did not change significantly with time and remained constant at 5 mg L^−1^. After the addition of the synthesized MA-PF127 nanomaterial, the dissolved oxygen content in the solution was significantly elevated, and the oxygen concentration increased nearly linearly with time, which indicates that the synthesized materials have significant catalytic activity in the presence of hydrogen peroxide. This leads to the generation of oxygen and facilitates the modification of the oxygen-depleted environment of the tumor. In Figure 3b, it can be seen that despite the elevated glutathione (GSH) content, a significant decrease in methylene blue (MB) content occurs even under neutral conditions Under acidic conditions, the specific absorbance values resulting from the degradation of MB are even lower, and the characteristics of the tumor microenvironment, such as lack of oxygen, acidity, and high GSH/H_2_O_2_ expression, limit photodynamic treatment. This is because the synthesized MA-PF127 nanomaterials generate a high amount of reactive oxygen species through a Fenton-like activity under acidic conditions, which is in accordance with our expected experimental results, demonstrating that the synthesized MA-PF127 nanomaterials possess similar chemodynamic activity to that of manganese tetroxide and have good potential to modulate the tumor microenvironment.

### 3.3. T_1_ MRI and Relaxivity Properties of Mn_3_O_4_-Ag_2_S-PF127 JNPs

Magnetic correlation measurements of the MA-PF127 JNPs were evaluated using an MRI scanner at 0.55 T.

**Figure 4:**
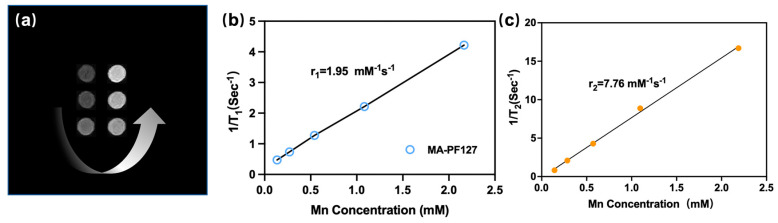
(**a**) The T_1_-weighted MR images at room temperature (**b**) The longitudinal relaxation rate of the MA-PF127 JNPs aqueous suspensions while varying the concentration of Mn. (**c**) The transverse relaxation rate of the MA-PF127 JNPs aqueous suspensions while varying the concentration of Mn.

As shown in Figure 4a, the MRI signal intensity was significantly enhanced with an increasing gradient of Mn concentration. The value of the relaxivities (relaxation rates of a solution change as a function of Mn concentrations), r_1_ and r_2_, of the MA-PF127 sample were measured through linearly fitting the spectral value of the sample relaxation time to the effective Mn concentration (mM). The calculated r_1_ and r_2_ were 1.95 mM^−1^s^−1^ and 7.76 mM^−1^s^−1^ (Figure 4b,c), respectively, which is significantly better than that of a series of previously reported Mn-based nanomaterials. Additionally, the ratio r_2_/r_1_ is 3.97, which is less than 5 [48], demonstrating that the synthesized MA-PF127 sample can potentially be used as a T_1_-MRI contrast agent.

### 3.4. Biocompatibility and Photothermal Therapy of Mn_3_O_4_-Ag_2_S-PF127 JNPs

Generally, quantum dots have attractive physical and chemical properties, such as tunable emission, superb light stability, broad Stokes shift, and high quantum yield. Ag_2_S quantum dots have been systematically investigated with emission ranging in the near-infrared (NIR) region, which can be divided into the first NIR window and the second NIR window, and have shown impressive potential in biomedical applications due to their low-energy emission and deeper tissue penetration with less damage to cells. In order to explore the function of the sole Ag_2_S part in the MA-PF127 NPs, the photothermal properties and efficiency of the synthesized MA-PF127 NPs were investigated using external stimuli from a 1064 nm NIR laser. A thermal infrared camera recorded the real-time temperature variation profile and images.

**Figure 5:**
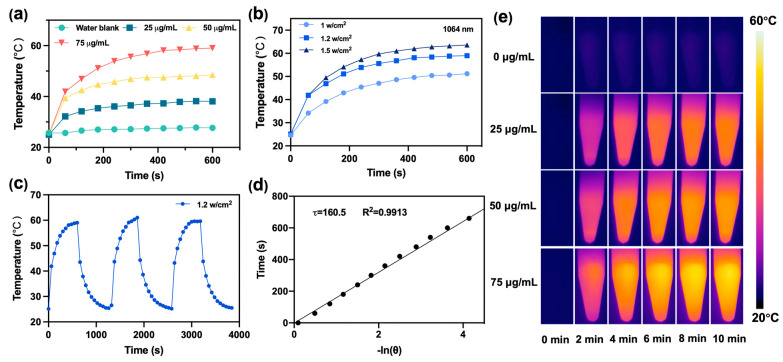
(**a**) The photothermal conversion profile of the MA-PF127 NPs at different concentrations (0, 25, 50, and 75 μg mL^−1^) with irradiation using a 1064 nm laser at 1.2 W cm^−2^ power intensity. (**b**) The photothermal conversion profile of the MA-PF127 NPs using variable laser power intensity (1, 1.2, and 1.5 W cm^−2^). (**c**) Cyclic photothermal stability tests of 75 μg mL^−1^ NPs solution under 1.2 W/cm^2^ laser irradiation. (**d**) The data from linear fitting time calculated from the cooling period with 75 μg mL^−1^ NPs solution under 1.2 W cm^−2^ laser irradiation (**e**) Photothermal images using an infrared camera.

In this study, we conducted experiments to evaluate the photothermal performance of MA-PF127 NPs at various concentrations under the intensity of 1.2 W cm^−2^. The results (Figure 5a) revealed that, when compared to the control group maintained at 25 °C, the MA-PF127 NPs at a high concentration (75 μg mL^−1^) exhibited a notable increment in temperature, reaching 59 °C within ten minutes of laser irradiation. This enhanced temperature variation also demonstrated a clear dependence on concentration. After that, we investigated the effect of different laser powers of the MA-PF127 NPs at 75 μg mL^−1^, as depicted in Figure 5b. It was observed that the temperature increased to 63 °C when exposed to laser irradiation at 1.5 W cm^−2^. Furthermore, analysis of the temperature curves through alternating the NIR laser on/off for three cycles revealed that the maximum temperature reached by the MA-PF127 NPs remained consistent over time (Figure 5c), indicating the excellent photothermal stability of the MA-PF127 material. Moreover, the photothermal conversion efficiency (η) of the MA-PF127 nanoparticles (NPs) was determined to be 53% using a formula derived from the cooling curve depicted in Figure 5d. Real-time images obtained from infrared thermography can enhance our comprehension of the photothermal performance of the MA-PF127 NPs at varying concentrations. As illustrated in Figure 5e, it is evident that the photothermal performance of the MA-PF127 NPs is contingent upon both concentration and time, showcasing their significant potential as photothermal agents.

**Figure 6:**
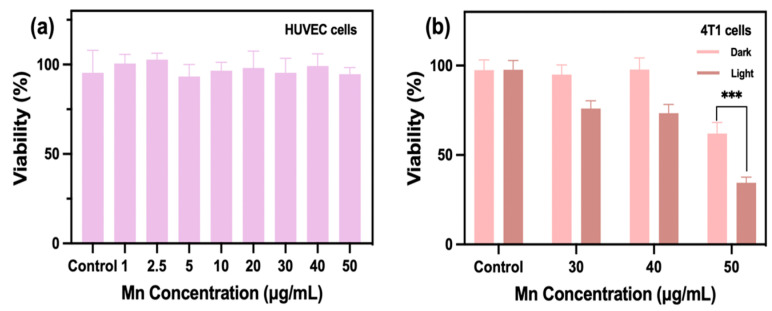
(**a**) Cell viabilities of HUVEC cells treated with different concentrations of the MA-PF127 NPs for 24 h. (**b**) Cell viabilities of 4T1 cells treated with the MA-PF127 NPs under 1064 nm laser light irradiation at 1.2 w cm^−2^ (*** *p* < 0.001).

To verify the potential medical applications of the MA-PF127 NPs, the human umbilical vein endothelial cells (HUVEC) were selected to be cultured with various concentrations of the MA-PF127 NPs to prove their biocompatibility. From Figure 6a, the specific range of chosen concentrations of the MA-PF127 NPs has absolute biosafety to normal human cells.

Based on the outstanding photothermal properties of the MA-PF127 NPs, in vitro toxicity at the cellular level was assessed (Figure 6b). Compared to normal human cells (HUVEC), the MA-PF127 NPs showed higher cytotoxicity when concentrations reached 50 μg mL^−1^, indicating that the nanoparticles can sensitively differentiate the neutral/acidic environments and may specifically kill the cancer cells. When the cells were seeded in a 96-well plate with materials irradiated with NIR light, the heat generated from the light via the photothermal transfer ability of the materials killed more cells through significantly decreasing cell viability, as shown in Figure 6b. In conclusion, the synthesized MA-PF127 JNPs can be used as a photothermal agent for further medical applications.

### 3.5. Antibacterial Activities

Herein, we also utilized the prepared JNPs as a potential antibacterial agent. The antibacterial properties of the synthesized Mn_3_O_4_-Ag_2_S JNPs were verified via treating Gram-negative bacteria (*Staphylococcus aureus*, *S. aureus*) and Gram-positive bacteria (*Escherichia coli*, *E. coli*) with different concentrations of the materials for 24 h, and bacterial survival was measured at OD_600 nm_ of the treated bacterial suspension using a UV spectrophotometer.

**Figure 7:**
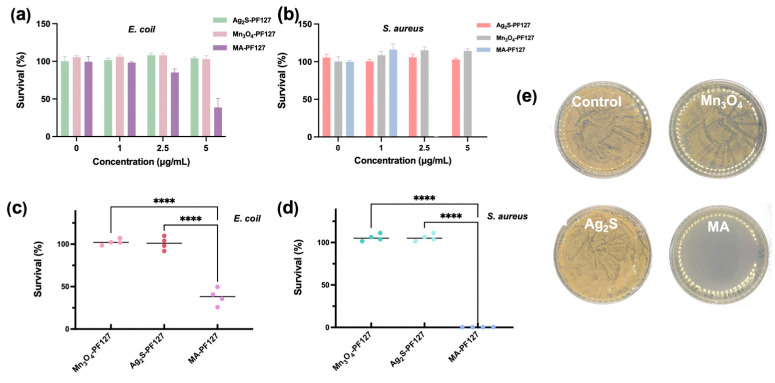
Antibacterial effect of the Mn_3_O_4_-PF127, Ag_2_S-Mn_3_O_4_-Ag_2_S, and MA-PF127 NPs at various concentrations (0, 1, 2.5, and 5 μg mL^−1^) of *E. coli* and *S. aureus*. The survival of *E. coli* (**a**) and *S. aureus* (**b**) in the solution containing materials at different concentrations. The survival of *E. coli* (**c**) and *S. aureus* (**d**) and the spread plate pictures of *S. aureus* (**e**) in the solution containing materials at 5 μg mL^−1^ (**** *p* < 0.0001).

As shown in Figure 7a,b, the antibacterial effect of MA-PF127 was more positive than that of Mn_3_O_4_-PF127 and Ag_2_S-PF127 at the same concentrations. With a linear increase in concentration, MA-PF127 showed noticeable improvement in killing bacteria at 2.5 μg mL^−1^ against *E. coli* and *S. aureus*. In addition, the antibacterial effect of MA-PF127 reached an aggressive level when the concentration was only 5 μg mL^−1^ compared to those of Mn_3_O_4_-PF127 and Ag_2_S-PF127 under the same conditions, as shown in Figure 7c,d. Additionally, the bactericidal effect of the synthesized materials was evaluated via agar plate counting experiments, and spread plate tests were performed using the remaining suspension of *S. aureus* with diverse materials at 5 μg mL^−1^, which can be seen in Figure 7e. The LB agar medium showed a clear and smooth surface without any bacteria, in contrast to the control group and other materials, indicating a strong antibacterial effect of the fabricated MA-PF127 NPs.

Ag ions are inherently antimicrobial, exhibit long-lasting bactericidal effects against several bacteria, such as *Staphylococcus aureus* and *Escherichia coli*, and are stable at high temperatures [49]. In addition, Mn^3+^ is a strong oxidant because of its high reduction potential (E0 = 0.51 V). Mn^3+^-containing materials have been reported to have antimicrobial activity, which may be attributed to Mn-induced oxidative damage. In addition, Mn^2+^ and Mn^3+^ are the two primary oxidation states of Mn in biological tissues. Mn^3+^ can be converted to Mn^2+^ under physiological conditions, producing reactive oxygen species (ROS) that accumulate during the transition. Excess ROS production above basal levels can oxidatively damage the membrane structure and genetic factors of bacteria, leading to bacterial death [50]. Moreover, Sulfur and its derivatives are known to have broad-spectrum antimicrobial properties and have been used effectively in treating dermatological and botanical diseases [51]. After surface modification with PF127, JNPs easily entered the biofilm, which increased the antibacterial activity and enhanced the bacterial uptake of the nanoparticles, which in turn killed the bacteria. Therefore, the enhanced antibacterial properties are attributed to the biocompatible coating and synergistic effect of the JNPs.

## 4. Conclusions

In summary, this article introduces an enhanced reversed-phase microemulsion technique for synthesizing heterogeneous structures comprising two or more components, incorporating an optimal template for the materials’ synthesis in analogous arrangements. Furthermore, the experimental outcomes validate the successful production of Mn_3_O_4_-Ag_2_S Janus nanomaterials, which possess exceptional T_1_-magnetic resonance imaging properties attributed to manganese as a good alternative to gadolinium chelates, which possess serious side effects, along with the photothermal characteristics of small-sized Ag_2_S, showcasing their promising potential for use as nanomedicine for cancer and bacterial treatment.

## Data Availability

Not applicable.
